# Integration of chromosomal microarray analysis and whole-exome sequencing for prenatal diagnosis of fetuses with cardiac ultrasound anomalies

**DOI:** 10.3389/fgene.2025.1668252

**Published:** 2026-01-05

**Authors:** Youlan Wu, Gui Chen, Fang Yang, Xiang Liu, Yawen Qiang, Renhua Wu, Fang Liu, Weisheng Cheng, Jing Yuan

**Affiliations:** 1 Prenatal Diagnostic Center, Department of Obstetrics and Gynecology, The First Affiliated Hospital of Anhui Medical University, Hefei, China; 2 Department of Obstetrics and Gynecology, NHC Key Laboratory of Study on Abnormal Gametes and Reproductive Tract, The First Affiliated Hospital of Anhui Medical University, Hefei, China; 3 Anhui Provincial Institute of Translational Medicine, Anhui Medical University, Hefei, China; 4 Department of Obstetrics and Gynecology, The First Affiliated Hospital of Anhui University of Science and Technology, Huainan, Anhui, China; 5 Xinjiang Second Medical College, Karamay, Xinjiang, China; 6 Department of Laboratory Medicine, The First Affiliated Hospital of Anhui Medical University, Hefei, China

**Keywords:** prenatal diagnosis, chromosomal microarray analysis, whole-exome sequencing, congenital heart disease, cardiac ultrasound

## Abstract

**Background:**

Congenital heart disease is among the most prevalent birth defects. This study aims to evaluate the clinical utility of chromosome microarray analysis (CMA) and whole-exome sequencing (WES) in prenatal diagnosis of genetic disorders in fetuses with cardiac ultrasound abnormalities.

**Methods:**

A retrospective cohort study analyzed 469 cases exhibiting fetal cardiac anomalies identified through prenatal ultrasound from November 2022 to July 2024. The study retrospectively assessed the patients' clinical features, observations, and pregnancy outcomes.

**Results:**

Out of the 469 cases meeting the inclusion criteria, conventional karyotyping identified chromosomal aneuploidies in 17 cases (3.62%). CMA identified pathogenic or likely pathogenic findings, including both aneuploidies and copy number variants, in 35 cases, yielding a detection rate of 7.46% (95% CI: 5.24%–10.21%). The incremental yield of CMA over karyotyping was 3.84%. WES was performed on 59 CMA-negative/variants of undetermined clinical significance cases, identifying pathogenic/likely pathogenic variants in 6/59 (10.17%; 95% CI 3.82%–20.87%), providing a cohort-level incremental yield of 1.28% (6/469).

**Conclusion:**

This study highlights the clinical significance of CMA and WES in the prenatal diagnosis of genetic disorders in fetuses presenting with cardiac ultrasound abnormalities. It affirms the robust utility of CMA and WES methodologies in prenatal diagnosis and genetic counseling.

## 1 Introduction

Congenital heart disease (CHD) encompasses structural or functional abnormalities in the heart that manifest at birth due to disruptions during embryonic or fetal development. It is a prevalent birth defect, with an incidence ranging from 7 to 22.9 per 1,000 live births and is prevalent in China (Xu and Shu, 2025). The etiology of CHD is multifaceted, involving a complex interplay of genetic and non-genetic factors (Pierpont et al., 2018). Genetic factors comprise chromosomal aneuploidy, chromosomal copy number variations, and single-gene mutations, while non-genetic factors include teratogenic exposures, maternal illnesses, maternal history of exposures, and infections (Blue et al., 2012). Genetic factors play a pivotal role in the pathogenesis of CHD. Chromosomal abnormalities or aneuploidy are detected in 8%–12% of CHD cases, chromosomal copy number variants in 3%–25% of cases, and single gene variants in known CHD-related genes in 3%–5% of cases. The likelihood of identifying a genetic anomaly is higher in non-isolated cases of CHD (Nees and Chung, 2020). Chromosomal aneuploidies, notably trisomy 21, trisomy 18, and trisomy 13, are prevalent in cases of CHD (Dykes et al., 2016). Copy number variations (CNVs) are significant contributors to CHD, often encompassing genes linked to CHD or critical for heart development. Single-nucleotide variants (SNVs) in relevant genes may also contribute to CHD pathogenesis (Simmons and Brueckner, 2017). Despite advancements in diagnosing and treating CHD (Kanneganti et al., 2023), the exploration of the association between CHD and chromosomal abnormalities remains constrained.

Karyotyping and fluorescence *in situ* hybridization (FISH) have traditionally been used to detect chromosomal aneuploidies and balanced translocations in prenatal cases of congenital heart disease. Nonetheless, these methods possess inherent limitations. Karyotyping is characterized by prolonged processing times and limited resolution, while FISH exhibits restricted probe coverage across chromosomes. In contrast, chromosomal microarray analysis (CMA) is adept at identifying abnormalities in chromosomal microfragments exceeding 100 kb, thereby enabling the detection of various associated syndromes arising from chromosomal microduplications and microdeletions (Levy and Wapner, 2018). CMA has gained prominence in prenatal diagnostics due to its ability to uncover these abnormalities. However, CMA is incapable of identifying balanced chromosomal rearrangements and SNVs, necessitating the integration of results with complementary tests for clinical interpretation. Beyond structural aberrations and CNVs, SNVs play a pivotal role in the genetic etiology of CHD. Whole-exome sequencing (WES) emerges as a valuable tool for pinpointing single-base variants and subtle genetic alterations at the exon level, thereby facilitating the diagnosis of genetic disorders that may elude detection through conventional cytogenetic approaches. WES has emerged as a powerful diagnostic tool with broad applications in clinical settings (Farwell et al., 2015; Taylor et al., 2015; Jin et al., 2017).

The integration of CMA and WES offers a thorough strategy for elucidating the genetic etiology of congenital heart defects in fetuses (M et al., 2024). This study aimed to assess the clinical utility of CMA and WES in identifying CNVs and SNVs in fetuses presenting with prenatal cardiac ultrasound abnormalities. The study also sought to compare diagnostic yields across various subgroups to enhance the understanding of the genetic underpinnings of ultrasound anomalies, thereby facilitating genetic counseling and informing clinical decision-making for cases involving prenatal fetal cardiac anomalies.

## 2 Materials and methods

### 2.1 Population

This retrospective cohort study, approved by the Ethics Committee of the First Affiliated Hospital of Anhui Medical University, enrolled 469 patients with prenatal ultrasound-detected cardiac abnormalities between November 2022 and July 2024. All participants underwent comprehensive genetic counseling and provided informed consent. Inclusion criteria comprised singleton pregnancy, suspected fetal CHD based on prenatal ultrasound findings, and access to genetic testing. Exclusion criteria included abnormal TORCH testing and multiple pregnancies. The gestational age at invasive procedure ranged from 17 to 33 weeks. Participants were referred to the prenatal diagnosis center for genetic counseling and testing. Couples who opted for prenatal CMA subsequently underwent invasive sampling. Cases with clinically significant CMA findings received comprehensive genetic counseling, whereas those with negative CMA results were further evaluated by WES. Parental follow-up testing was recommended for all cases with pathogenic/likely pathogenic copy-number variants or variants of uncertain significance to ascertain inheritance. Data on maternal age, gestational age, test results, associated ultrasound findings, and pregnancy outcomes were retrospectively collected through medical record review and telephone follow-up. The study received approval from the Ethics Committee of the First Affiliated Hospital of Anhui Medical University, and written informed consent was obtained from all participants.

### 2.2 Definition and classification of sonographic indicators

Considering that referrals during late gestation (greater than 28 weeks) are associated with complex anomalies, this study conducted a statistical categorization of fetal cardiac ultrasound findings based on gestational age. The first category, soft markers, was defined as including isolated echogenic intracardiac focus (EIF). The second category, structural congenital heart disease, was classified as follows: septal defects included ventricular septal defects and atrioventricular septal defects. However, due to their typically benign clinical course, isolated small muscular ventricular septal defects and isolated atrial septal defects were defined as risk factors and excluded from the “isolated structural CHD” group for genetic detection rate analysis. Conotruncal defects included tetralogy of Fallot, transposition of the great arteries, double outlet right ventricle, and truncus arteriosus. Left ventricular outflow tract (LVOT) obstruction included hypoplastic left heart syndrome, critical aortic stenosis, and coarctation of the aorta. Additional classifications comprised heterotaxy syndrome/laterality defects, and other CHDs, including valvular abnormalities and cardiomyopathies. The third category, extracardiac anomalies, included structural abnormalities of the brain, urinary system, digestive system, musculoskeletal system, and facial defects. The fourth category, designated as “other,” included tricuspid regurgitation and other risk factors.

### 2.3 Karyotyping

Amniotic fluid was centrifuged at 1,500 rpm for 10 min to isolate cells, which were then cultured under standardized conditions. Cells were harvested based on their growth status post-culture. After routine slide preparation, Giemsa banding (G-banding) was conducted. 20 metaphase cells were microscopically examined, and at least five karyograms were recorded with a resolution of at least 320 bands. All procedures strictly adhered to the guidelines outlined in the International System for Human Cytogenetic Nomenclature (ISCN) (ISCN, 2024). The median turnaround time (TAT) was 21 days for karyotyping.

### 2.4 Chromosomal microarray analysis

The Affymetrix CytoScan™ 750K array (Applied Biosystems, USA) was used for chromosomal microarray analysis following the manufacturer’s instructions. In brief, genomic DNA from amniotic fluid cells was extracted using the Micro Sample Genomic DNA Extraction Kit (TIANGEN, China). The extracted DNA was digested by the *Nsp* I restriction enzyme, followed by ligation of Nsp I adapters to the digested fragments using T4 DNA ligase, and subjected to amplification through PCR to generate fragments ranging from 150 to 2000 bp. Subsequently, the PCR products were purified and subjected to a PCR fragmentation step, resulting in fragments ranging from 25 to 125 base pairs (bp). The fragmented products then underwent a labeling reaction catalyzed by Terminal Deoxynucleotidyl Transferase (TdT), followed by hybridization and denaturation on the Affymetrix CytoScan™ 750K array at 50 °C for 16–18 h. Finally, the array was washed, stained, and scanned. Data analysis was performed using Affymetrix Chromosome Analysis Suite (ChAS) v4.4, and the results were mapped to the Genome Reference Consortium Human Build 37 (GRCh37/hg19) genome. Concurrently, the UCSC genome database was queried to obtain the GRCh38/hg38 coordinate system (Table 1; Supplementary Table S1). Quality control parameters included a median absolute pairwise difference (MAPD) ≤ 0.25 and SNPQC >12. Valid CNV reporting thresholds are ≥500 kb for deletions and ≥1,000 kb for duplications.

**TABLE 1:** List of pathogenic and potentially pathogenic CNVs identified by CMA.

Karyotype	CMA	GRCh37/hg19 coordinates	GRCh38/hg38 coordinates	Size	Inheritance	Classification	Cases	Phenotype
47,U,+21	Trisomy 21	-	-	-	*de novo*	P	7	Cardiovascular dysplasia, echogenic intracardiac focus, choroid plexus cyst, nasal dysplasia
47,U,+18	Trisomy 18	-	-	-	*de novo*	P	5	Complete atrioventricular septal defect, choroid plexus cyst, cardiovascular dysplasia
47,U,+13	Trisomy 13	-	-	-	*de novo*	P	1	Beak nose, microphthalmia, maxillary dysplasia, ventricular septal defect, atrial septal defect, aortic dysplasia
46,U	Mosaic trisomy 3	-	-	-	*de novo*	P	1	Tricuspid regurgitation, increased bowel echogenicity, fetal growth restriction
45,X/46,XY	45,X/46,XY mosaicism	-	-	-	*de novo*	P	1	Mild tricuspid regurgitation
46,U	dup 14q32.2q32.33 del 21q22.3	chr14:96863160–107284437 chr21:45217753–48093361	The region corresponds to several discrete intervals in GRCh38 (details in Supplementary Table S1) chr21:43797872–46673449	10.4 Mb 2.8 Mb	*de novo* *de novo*	P P	1	Echogenic intracardiac focus, midline intracranial cystic structure
46,U	del 7q11.23	chr7:72701099–74154209	chr7:73287097–74739874 chr7:73375011–73375166	1.4 Mb	*de novo*	P	1	Ventricular septal defect, short femur
46,U,der (4)?	del 4p16.3p15.33 dup 4q27q35.2	chr4:68346–15123217 chr4:121879847–190957460	chr4:68454–15121593 chr4:120958692–190036305	15.0 b 69.0 Mb	*de novo* *de novo*	P P	1	Echogenic intracardiac focus, nasal bone hypoplasia
46,U	del 4q35.2 dup 8p23.3p12	chr4:189875605–190957460 chr8:158049–33013307	chr4:188954451–190036305 chr8:208049–33155789 chr8_KI270811v1_alt:17104–17141	1.0 Mb 32.8 Mb	*de novo* *de novo*	VUS P	1	Cardiovascular dysplasia
46,U	del 22q11.21	chr22:18648856–21464764	chr22_KI270731v1_random:120986–121187 chr22:18429208–18659564	2.8 Mb	*de novo*	P	1	Persistent truncus arteriosus, ventricular septal defect, aortic override
46,U,inv (9)(p12q13)	dup 1q21.1q21.2	chr1:146586250–147995251	chr1:147114668–148459811 chr15:39894315–39894344 chr1:145490428–145490458 chr8:38661816–38661844 chr1:145475939–145475974	1.4 Mb	*de novo*	P	1	Urinary dysplasia, nasal bone dysplasia/absence, tricuspid regurgitation
46,U	del 7q11.23	chr7:72664089–74154527	chr7:73250057–74740192 chr7:73375011–73375166	1.4 Mb	*de novo*	P	1	Echogenic intracardiac focus
46,U	del 6q25.1	chr6:149390350–152454473	chr6:149069214–152133338	3.06 Mb	*de novo*	P	1	Tricuspid regurgitation, short long bones
45,X (70)/46,X,+mar (30)	del Xp11.21q23 del Xp22.33p11.21 del Xq23q28	chrX:168552–55476636 chrX:55414457–112956400 chrX:112753369–155233098	The region corresponds to several discrete intervals in GRCh38 (details in Supplementary Table S1)	57.5 Mb 55.3 Mb 42.4 Mb	*de novo* *de novo* *de novo*	LP LP LP	1	Tricuspid regurgitation
46,U	del 15q11.2	chr15:22770422–23276605	chr15:22596491–23102646 chr15:22770568–22770734	506 Kb	*de novo*	P	2	Fetal head circumference below the gestational age reference, mild tricuspid regurgitation, increased bowel echogenicity
46,U	dup 17p11.2q12	chr17:21298976–34865813	chr17:37970531–37971356 chr17_KI270857v1_alt:2156539–2156574	13.56 Mb	*de novo*	P	1	Bilateral hydronephrosis, mild tricuspid regurgitation
46,U	del 16p13.11p12.3	chr16:15241281–18242713	chr16:15147424–18148856	3 Mb	*de novo*	LP	1	Aortic valve stenosis with poststenotic dilatation of the ascending aorta, middle cerebral artery with elevated peak systolic velocity and prolonged acceleration time
46,U	del 22q12.2	chr22:29987878–30029330	chr22:29591889–29633341	41 Kb	*de novo*	LP	1	Nuchal lymphatic hygroma, mild tricuspid regurgitation
46,U	del 6q27	chr6:169711386–170914297	chr6:169311291–170605209	1.20 Mb	*de novo*	P	1	Reduced transverse cerebellar diameter, echogenic intracardiac focus
46,U	del Xp22.33p22.31	chrX:168552–7685339	The region corresponds to several discrete intervals in GRCh38 (details in Supplementary Table S1)	7.52 Mb	*de novo*	P	1	Tricuspid regurgitation, short long bones
46,U	del 8p23.1	chr8:8093066–11935465	chr8:8235544–12077956	3.84 Mb	*de novo*	P	1	Cardiac axis deviation to the right, echogenic intracardiac focus, left congenital diaphragmatic hernia
46,U	del 16p11.2p11.2	chr16:28834571–29001626	chr16:28823250–28990305	0.17 Mb	*de novo*	P	1	Ventricular septal defect, fetal growth restriction
46,U	del 17q12	chr17:34842505–36104986	chr17:36486661–37744995	1.26 Mb	*de novo*	P	1	Bilateral hyperechoic kidneys, narrowing of the pulmonary artery, mild to moderate tricuspid regurgitation, no discernible anal target was identified
46,U	del 11q24.1q25 dup q23.3q24.1	chr11:123301470–134667626 chr11:118495644–123065714	chr11:123430762–134797732 chr11:118624928–123195006	11.37 Mb 4.57 Mb	*de novo* *de novo*	P VUS	1	Left foot abnormality , left ventricle appearing mildly hypoplastic , short left foot bone , poorly visualized phalanges of the left foot

Out of 469 fetuses with cardiac ultrasound abnormalities, 66 chromosomal abnormalities were identified through CMA, resulting in a detection rate of 14.07%. Notably, pathogenic or likely pathogenic chromosomal abnormalities were detected in 35 cases, corresponding to a detection rate of 7.46% (95% CI: 5.24%–10.21%).

The CNVs were systematically assessed for their clinical relevance after quality control measures were applied. This evaluation involved a comprehensive review of pertinent scientific literature and various public databases, including the UCSC Genome Browser, PubMed, Database of Genomic Variants, OMIM, DECIPHER, and ClinGen Dosage Sensitivity Maps. The CNVs were categorized according to the guidelines established by the American College of Medical Genetics and Genomics (ACMG) and Prenatal Diagnosis Group, Society of Medical Genetics, Chinese Medical Association (Riggs et al., 2020; Prenatal and Liu, 2023). These categories include pathogenic (P) CNVs, likely pathogenic (LP) CNVs, variants of undetermined clinical significance (VUS), possibly benign (LB) CNVs, and benign (B) CNVs. The median TAT was 18 days for CMA.

### 2.5 Whole-exome sequencing

Genomic DNA from the fetus and both parents was extracted with the QIAamp DNA Blood Mini Kit, followed by fragmentation using sonication. Library preparation and exome capture were performed using the KAPA HyperExome V2 Kit. Sequencing was conducted on the MGISEQ-T7 (MGI Tech Co., Ltd., Shenzhen, China) high-throughput sequencer with 2 × 150 bp paired-end reads. The mean on-target depth was 200×, with >95% of targeted bases covered at ≥20×. Fastp filtering software was employed to eliminate adapter sequences and low-quality reads (Chen, 2023). Qualified reads were aligned to the human reference genome (GRCh37/hg19) through Sentieon to produce alignment files. Subsequently, variant calling was executed using Sentieon after sorting and duplicate marking (Freed et al., 2022). Identified variants undergo detailed annotation using the VEP (Variant Effect Predictor) software (Howe et al., 2021), which encompasses information such as population frequency, associated genes and transcript positions, sequence conservation, amino acid alterations, position within the protein structure, predicted impact on protein function, and annotations from public databases. Putative pathogenic variants were validated by Sanger sequencing. Inheritance patterns were determined by segregation analysis in trios. Ultimately, variant pathogenicity was evaluated and interpreted in accordance with the ACMG standards and guidelines (Richards et al., 2015). The median turnaround time (TAT) was 21 days for karyotyping.

### 2.6 Statistical analyses

Chromosomal abnormalities and gene mutation detection rates were compared between isolated and non-isolated groups using either the chi-square test or Fisher’s exact probability method. A significance level of *P* ≤ 0.05 was applied to determine statistical significance—95% confidence intervals (CIs) for proportions calculated using the Clopper-Pearson exact method. Odds ratios (ORs) with 95% CIs were calculated for group comparisons.

## 3 Results

### 3.1 Patient characteristics

**FIGURE 1:**
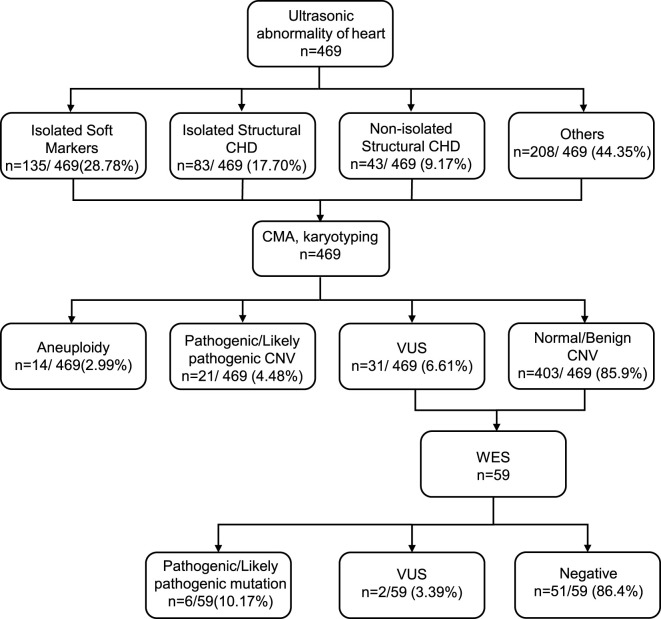
Flow chart of the study.

From November 2022 to July 2024, 469 pregnant women with prenatal cardiac ultrasound anomalies underwent invasive prenatal testing at the Prenatal Diagnostic Center (Figure 1). The average age of these women was 31.1 years, with a range of 18–45 years. The mean gestational age at the time of the invasive procedure was 24.47 weeks, with a range of 17–33 weeks. Among the 469 pregnancies included, 20 (4.3%) were referred for invasive testing due to a high-risk non-invasive prenatal testing (NIPT) result.

**FIGURE 2:**
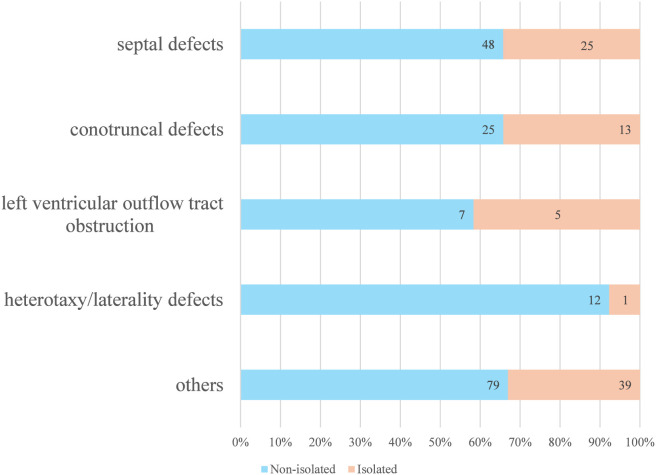
Distribution of Cases by Phenotypic Subgroup and Isolation Status. This stacked bar chart illustrates the proportional distribution of cases across different phenotypic categories. Each bar is further stratified to show the proportion of isolated and non-isolated cases within each subgroup.

Among the 469 fetuses that underwent prenatal testing, 218/469 (46.5%) had only cardiac ultrasound abnormalities, of which 135/469 (28.8%) were isolated soft markers (EIF), 83/469 (17.7%) were isolated structural CHD, Isolated structural CHD comprised septal defects (n = 25), conotruncal defects (n = 13), LVOT (n = 5), heterotaxy/laterality defects (n = 1), and other CHD (n = 39) (Figure 2). 251/469 (53.5%) exhibited cardiac ultrasound abnormalities with other system anomalies. The most common cardiac ultrasound abnormality was LVOT. In cases where cardiac ultrasound abnormalities coexisted with other issues, the predominant findings included EIF in 90 cases (35.86%) and ventricular septal defects in 21 cases (8.37%). Other associated systemic anomalies included urinary anomalies in 64 cases (26.5%), brain anomalies in 53 cases (21.12%), nuchal translucency thickening in 31 cases (12.35%), facial defects in 22 cases (8.76%), fetal biometrics lagging behind gestational age in 20 cases (7.97%), digestive abnormalities in 19 cases (7.57%), musculoskeletal developmental abnormalities in 17 cases (6.77%), and excess amniotic fluid in 11 cases (4.38%). Most of these chromosomal abnormalities were *de novo* variants, in contrast to 15/73 (20.55%) that were inherited.

### 3.2 CMA test results

A total of 469 fetuses exhibiting cardiac ultrasound abnormalities underwent CMA, resulting in the detection of 66 chromosomal abnormalities, yielding a detection rate of 14.07% (95% CI: 11.08%–17.57%). Specifically, pathogenic/likely pathogenic (P/LP) chromosomal abnormalities were identified in 35 cases, corresponding to a detection rate of 7.46% (95% CI: 5.24%–10.21%). Among these cases, there were 14 instances of aneuploidy, encompassing 7 cases of trisomy 21, 5 cases of trisomy 18, 1 case of trisomy 13, 1 case of mosaic trisomy 3, and 1 case of 45,X/46,XY mosaicism. Furthermore, pathogenic or probably pathogenic chromosomal CNVs were observed in 21 fetuses, including common cardiovascular system-related syndromic microdeletions, involving one case of 22q11.2 deletion syndrome, two cases of 7q11.23 deletion syndrome, and one case of 8p23.1 deletion syndrome, all confirmed as *de novo* mutations (Table 1). Additionally, 31 fetuses had reported variants judged to be VUS, while CMA results for 403 fetuses showed no abnormalities within the range. In this cohort, all 469 fetuses underwent both karyotyping and chromosomal microarray (CMA). Karyotyping identified aneuploidies and large fragment copy number variants (CNVs) in 17 fetuses, yielding a detection rate of 3.62%. CMA demonstrated a significantly higher detection rate of 14.07%, representing an incremental yield of 10.45 percentage points over karyotyping, which is attributed to its ability to identify both numerical chromosomal abnormalities and submicroscopic deletions/duplications.

### 3.3 WES test results

WES was performed in 59/403 (14.64%) eligible CMA-negative/VUS cases, resulting in the detection of eight variants. P/LP variants were identified in 10.17% (6/59; 95% CI: 3.82%–20.87%) of the fetuses, with 66.7% (4/6) in the non-isolated group and 33.3% (2/6) in the isolated group (Table 2). In summary, the diagnostic yield of WES testing was 10.17% for fetuses presenting chromosomal aneuploidy or P/LP CNVs that were not identified through CMA.

**TABLE 2:** List of pathogenic and Likely pathogenic variants identified by WES.

Variant	Interpretation	Inheritance	Phenotype
PMS2:c.1579_1580del (p.R527Gfs*14)	Pathogenic (PVS1+PS4_P + PM2_P)	Maternal	Echogenic intracardiac focus in left ventricle; Aberrant right subclavian artery
PTPN11:c.236A>G (p.Q79R)	Pathogenic (PS2+PS3+PS4+PM2+PP1_S + PP2+PP3)	Paternal	Echogenic intracardiac focus in left ventricle; pleural effusion
APOB:c.10579C>T (p.R3527W)	Pathogenic (PS3_P + PS4+PM5+PP1_S + PP3)	Maternal	Ventricular septal defect; aortic override; pulmonary stenosis
MYH7:c.2605C>T (p.R869C)	Likely pathogenic (PM1+PM2_P + PM5+PP1+PP3)	Maternal	Situs inversus; Small perimembranous ventricular septal defect (VSD), mild ventriculomegaly
GPC3:c.888G>A (p.W296*)	Likely pathogenic (PVS1+PM2_P)	Maternal	Echogenic intracardiac focus
LDLR:c.599T>G (p.F200C)	Likely pathogenic (PS3+PS4_P + PM2)	Maternal	Mild tricuspid regurgitation; abdominal ascites

### 3.4 Gestational age and phenotypic differences in genetic variant detection

Based on the comprehensive analysis of chromosomal microarray and whole-exome sequencing results from 469 fetuses with abnormal cardiac ultrasound findings, significant associations were observed between variant detection rates and specific phenotypic characteristics. The overall variant detection rate was 15.3% (95% CI: 11.9%–19.2%) in the ≤28 weeks cohort (n = 405) and 17.2% (95% CI: 8.9%–28.7%) in the >28 weeks cohort (n = 64), with no statistically significant difference between gestational age groups (P = 0.687). Notably, the presence of extracardiac anomalies emerged as the strongest predictor of genetic variants. In the ≤28 weeks group, non-isolated structural congenital heart disease (CHD) demonstrated significantly higher detection rates (32.4%) compared to both isolated structural CHD (12.3%, P = 0.016) and isolated soft markers (5.8%, P < 0.001). This pattern was more pronounced in the >28 weeks group, where non-isolated structural CHD cases showed a 44.4% detection rate compared to 0% in both isolated groups (P < 0.05 for both comparisons). No significant differences were observed between gestational age groups in the detection rates of pathogenic/likely pathogenic variants (7.9% vs. 9.4%, P = 0.632) or variants of uncertain significance (7.7% vs. 7.8%, P = 0.968) (Table 3).

**TABLE 3:** CMA and WES results for isolated and non-isolated groups.

Gestational age group	Ultrasound phenotype	P/LP	VUS	Total	Variant detection rate	95% CI
≤28 weeks (n = 405)	Isolated soft markers (n = 121)	1	6	7	5.8%	2.4%–11.6%
	Isolated structural CHD (n = 73)	4	5	9	12.3%	5.8%–22.1%
	Non-isolated structural CHD (n = 34)	7	4	11	32.4%	17.4%–50.5%
	Others (n = 177)	19	16	35	19.8%	14.2%–26.4%
	Total (n = 405)	31	31	62	15.3%	11.9%–19.2%
>28 weeks (n = 64)	Isolated soft markers (n = 14)	0	0	0	0.0%	0.0%–22.9%
	Isolated structural CHD (n = 10)	0	0	0	0.0%	0.0%–30.8%
	Non-isolated structural CHD (n = 9)	2	2	4	44.4%	13.7%–78.8%
	Others (n = 31)	4	3	7	22.6%	9.6%–41.1%
	Total (n = 64)	6	5	11	17.2%	8.9%–28.7%

These findings establish extracardiac anomalies (OR = 3.85, 95%CI: 1.85–8.02) as a crucial independent risk factor for fetal genetic variants, while suggesting that gestational age has limited impact on overall detection rates, though it may influence risk distribution across specific phenotypic categories. Furthermore, in the >28 weeks cohort, likely due to the enrichment of severe cases and the inherent instability of small sample sizes, the point estimate of the variant detection rate was higher in non-isolated CHD (44.44% vs. 32.4% in the ≤28 weeks group). Although the detection rate was 0% for isolated anomalies in this late-gestation group, the confidence intervals suggest that the underlying risk cannot be overlooked.

### 3.5 Pregnancy outcomes

Patients who continued their pregnancies were monitored for 3 months and 1 year postpartum. Among the 35 fetuses identified by CMA with chromosomally pathogenic or possibly pathogenic (P/LP) CNVs, 54.3% (19/35) chose to terminate pregnancy, 20% (7/35) had no complications, and the outcomes for the remaining nine were unknown due to loss to follow-up. Among the six fetuses who underwent prenatal WES for P/LP variant detection, one was terminated, one was born prematurely with pleural effusion and exhibited respiratory and feeding issues, while the other four had no complications. A premature infant with pleural effusion and respiratory and feeding issues was diagnosed with overriding aorta and pulmonary stenosis through postnatal ultrasound and clinical evaluation. For fetuses with negative/VUS CMA and WES results, the vast majority (>90%) of pregnancies were continued, resulting in live births without reported major neonatal complications at the time of follow-up. Two pregnancies resulted in spontaneous abortion after fetal demise, one case was postnatally confirmed to have a ventricular septal defect by imaging, seven cases were delivered preterm, and ten pregnancies were terminated due to complex cardiac anomalies and/or concurrent malformations in other organ systems. The outcomes of some cases remained unknown due to loss to follow-up. No patients pursued additional genetic confirmation post-birth.

## 4 Discussion

Chromosomal abnormalities, single and polygenic genetic defects, and environmental factors are known contributors to CHD. The genetic basis of CHD is intricate, with current prenatal genetic diagnostics primarily targeting chromosomal and single-gene disorders. However, the full phenotypic range of many genetic disorders during fetal development remains elusive. Structural anomalies identified through prenatal ultrasound may serve as early indicators of CHD. Soft markers on ultrasound, nonspecific findings observed during mid-pregnancy scans, are typically transient and benign. Nevertheless, they may signal an elevated risk of fetal chromosomal abnormalities (Hu et al., 2021). Notably, cardiac strong echoes, a soft marker detected in fetal ultrasound at approximately 5% (Freed et al., 2022), are often associated with a heightened likelihood of structural cardiac defects (Chiu et al., 2019). Research indicates that these fetal heart echoes may indicate significant conditions, such as congenital heart disease, and may also hint at the presence of chromosomal aberrations or genetic mutations (Lorente et al., 2017; Song et al., 2021). This retrospective analysis encompassed 469 cases of prenatal cardiac ultrasound abnormalities, some of which exhibited soft ultrasound markers, such as EIF.

Our study revealed a 7.46% detection rate of P/LP using CMA for fetal cardiac ultrasound abnormalities, encompassing aneuploidy in 2.99% and significant CNVs in 4.47%. CMA exhibited a significantly higher detection rate of aneuploidies and CNVs compared to traditional karyotyping. In cases where CMA yields negative results or detects VUS, WES shows an additional diagnostic rate of 10.17% in this subgroup and 1.28% in the entire cohort. These rates are associated with single-gene mutations or rare variants, potentially leading to complex heart abnormalities identified through WES. These results underscore the efficacy of integrating CMA with WES to uncover genetic abnormalities in instances of fetal cardiac ultrasound anomalies. Our stratified analysis revealed that the diagnostic yield was highly dependent on the phenotype. The diagnostic yield of both CMA and WES was highest in fetuses with cardiac abnormalities accompanied by extracardiac anomalies, a finding consistent with previous reports (Sagi-Dain et al., 2021; Yin et al., 2025), and lowest in those with isolated soft markers. This demonstrates the particular significance of CMA in cases of CHD coexisting with extracardiac anomalies. The detection rate in our study, albeit lower than in previous research, may be attributed to variations in inclusion and exclusion criteria (Sun et al., 2024; Xing et al., 2022). Our detection rate of aneuploidies and pathogenic CNVs is lower than previously reported rates of 14.3% (84/586) (Xing et al., 2022). This discrepancy may be attributed to two reasons: first, 31 CNVs (6.6%, 31/469) classified as VUS in our cohort, were not subjected to further pathogenicity assessments, including family segregation analysis, phenotypic correlation, or updating our local genomic databases; and most importantly, the study’s inclusion of 34 fetuses (5.8%, 34/586) with trisomy 21, 18, or 13, typically identified through non-invasive prenatal test (NIPT) and prioritized for invasive prenatal diagnosis-may have contributed to the lower detection rate, as these cases were excluded from our study population. Notably, our study cohort encompassed fetuses with identified soft markers, a factor often overlooked. The detection rate of chromosomal abnormalities was notably higher in fetuses exhibiting ultrasound abnormalities in the heart alongside other systemic abnormalities, emphasizing the importance of prenatal diagnosis in these cases.

CMA is a high-resolution technology capable of identifying chromosomal structural variants such as microdeletions and microduplications, commonly linked to congenital heart disease development. WES focuses on coding regions to detect single-gene mutations or rare variants that may cause complex heart abnormalities. The integration of these two methodologies enhances the precision with which genetic underpinnings associated with fetal cardiac anomalies can be identified. This synergy is especially crucial in cases lacking evident chromosomal structural anomalies, where the genetic insights provided by WES play a pivotal role in diagnosis. The integration of CMA and WES has emerged as a significant focus in clinical research and practice concerning the diagnosis of prenatal fetal cardiac ultrasound abnormalities. These abnormalities often serve as early indicators of congenital heart disease. Nevertheless, conventional ultrasound assessments may be constrained in identifying complex cardiac conditions or those influenced by genetic factors. By combining CMA and WES, a more comprehensive genetic evaluation of fetal cardiac anomalies can be achieved, thereby enhancing diagnostic precision.

Our study has several limitations, most notably the potential for selection bias in the WES cohort. This bias arose from multiple factors: the substantial cost of testing, delays in CMA reporting that led some parents to decline additional invasive procedures, and the decision by some to terminate the pregnancy based on adverse ultrasound findings alone. Collectively, these factors contributed to the acquisition of WES in only 59 of the 403 CMA-negative cases. Our study incorporates clinically justified selection biases at both the institutional and patient levels. At the institutional level, the targeted use of WES was driven by a high-risk enrichment strategy, the rationale for a tiered diagnostic approach, and considerations of cost-effectiveness. At the patient level, factors such as the substantial cost of testing, delays in CMA reporting leading some parents to decline additional invasive procedures, and decisions to terminate pregnancies based solely on adverse ultrasound findings further shaped the cohort. These elements collectively created a pronounced conflict between prolonged test turnaround times and gestational age limits for termination. When genetic results became available late in gestation, families were often compelled to confront complex late-term termination decisions, significantly amplifying their psychological and social burden.

Statistically, in the >28 weeks gestation cohort, the point estimate for the detection rate of variants in non-isolated CHD was notably higher than that in the ≤28 weeks group. However, this observation should be interpreted with caution, as it may reflect both an increased severity of referred cases and the instability inherent in small sample sizes. Conversely, while a 0% detection rate was observed for isolated anomalies in the >28 weeks group, the upper limit of the confidence interval suggests that potential risk cannot be ruled out. This statistical nuance underscores the importance of considering confidence intervals rather than relying solely on point estimates, particularly in underpowered subgroups. In this study, WES identified a fetus carrying a pathogenic PMS2 variant, with ultrasound findings demonstrating both an echogenic intracardiac focus and an aberrant right subclavian artery. However, PMS2-related syndromes primarily confer cancer predisposition in childhood or adulthood (Herkert et al., 2011), without established causality to specific congenital cardiac or vascular anomalies. We therefore consider the sonographic findings likely coincidental rather than pathogenically linked to the PMS2 variant. This case highlights the interpretive challenges regarding incidental or secondary findings in prenatal WES applications.

The combination of CMA and WES improved the diagnostic yield for fetuses with cardiac ultrasound abnormalities. This integrated approach is particularly valuable in cases with inconclusive routine ultrasound findings and demonstrates clear clinical necessity for fetuses with CHD accompanied by extracardiac anomalies. This combined genetic testing approach has substantial clinical significance, as it not only enables precise prediction of fetal cardiac development but also establishes a robust foundation for subsequent clinical management and genetic counseling of families. Thus, the combination of CMA and WES in the assessment of prenatal fetal cardiac ultrasound anomalies exhibits considerable potential and utility, offering comprehensive support for early diagnosis, intervention, and evaluation of familial genetic risk.

## 5 Conclusion

A cohort study was conducted to assess the clinical utility of CMA and WES in prenatal genetic diagnosis of fetuses presenting cardiac ultrasound abnormalities. Findings indicated a notably higher detection rate of chromosomal abnormalities in fetuses with cardiac ultrasound abnormalities accompanied by extracardiac anomalies compared to those without extracardiac anomalies. The combined application of CMA and WES significantly enhances the diagnostic yield for fetal cardiac ultrasound abnormalities, underscoring the recommendation for their concurrent use in prenatal diagnostics. Notably, particular emphasis is advised for fetuses exhibiting cardiac ultrasound abnormalities in conjunction with extracardiac anomalies, particularly when accompanied by other structural irregularities or soft markers.

## Data Availability

The data presented in this article are not readily available because of ethical and privacy restrictions. Requests to access the data should be directed to the corresponding authors.
